# Novel Computerized Method for Measurement of Retinal Vessel Diameters

**DOI:** 10.3390/biomedicines5020012

**Published:** 2017-03-27

**Authors:** Hichem Guedri, Mariem Ben Abdallah, Fraj Echouchene, Hafedh Belmabrouk

**Affiliations:** 1 Electronics and Microelectronics Laboratory, Physics Department, Faculty of Sciences, Monastir University, Monastir 5019, Tunisia; mariembenabdallah3@gmail.com (M.B.A.); frchouchene@yahoo.fr (F.E.); Hafedh.Belmabrouk@fsm.rnu.tn (H.B.); 2 Department of Physics, College of Science AlZulfi, Majmaah University, Majmaah 15341, Saudi Arabia

**Keywords:** active contour, blood vessel diameter, Douglas-Peucker algorithm, eye diseases, retinal image

## Abstract

Several clinical studies reveal the relationship between alterations in the topologies of the human retinal blood vessel, the outcrop and the disease evolution, such as diabetic retinopathy, hypertensive retinopathy, and macular degeneration. Indeed, the detection of these vascular changes always has gaps. In addition, the manual steps are slow, which may be subjected to a bias of the perceiver. However, we can overcome these troubles using computer algorithms that are quicker and more accurate. This paper presents and investigates a novel method for measuring the blood vessel diameter in the retinal image. The proposed method is based on a thresholding segmentation and thinning step, followed by the characteristic point determination step by the Douglas-Peucker algorithm. Thereafter, it uses the active contours to detect vessel contour. Finally, Heron’s Formula is applied to assure the calculation of vessel diameter. The obtained results for six sample images showed that the proposed method generated less errors compared to other techniques, which confirms the high performance of the proposed method.

## 1. Introduction

Medical imaging encompasses methods for imaging human organs; this advanced technology makes it possible to improve the diagnosis and effective treatments of these organs [1,2,3,4]. Furthermore, the use of precise diagnosis and monitoring instruments can more quickly take into account and review various medical conditions and health events in clinical examinations [5,6].

Blood vessels play a pivotal role in the discovery, the observation, and the diagnosis of some medical conditions [7,8,9,10]. For example, retinal vessels may be affected by certain diseases such as diabetic retinopathy, hypertensive retinopathy, and macular degeneration [7,8]. They affect the structural characteristics of the blood vessels of the human retina. Therefore, the study and the analysis of the vessel geometric characteristics such as vessel diameter, branch angles, and branch lengths have become the basis of medical applications related to early diagnosis and effective monitoring of these diseases.

Therefore, this paper will seek to explore and identify a tool to measure the blood vessel diameter in retinal images and to acquire a computerized system that can automatically predict the change in the vessels width.

There are many methods used to detect blood vessel diameters. Kumar et al. [11] have introduced an automatic method for measuring the vessel diameter; this method is based on the Linear Discriminate Analysis (LDA). Bhuiyan et al. [12] have proposed a technique for measuring blood vessel width; this method is based on the vessel edge and centerline, in addition to the image gradient segmentation technique (ARG) for the vessel edge’s detection. Furthermore, it is based on the unsupervised texture classification method for obtaining the vessel centerline. At the final step, a mask rotation invariant was applied for center-line pixels, whose center corresponds to the pixels’ position. The potential pixels were drawn from the edge image using a continuous increment from lower to higher distance and orientation. El Abbadi et al. [13] have suggested a strategy to identify the retinal blood vessel diameter based on mask creation and specifically to measure the blood vessel diameter. Gao et al. [14,15] have proposed another method to measure the blood vessel diameter, which incorporates the use of a tracking technique. Twin Gaussian functions are introduced for modeling the distribution of a gray-level profile over a vessel cross section. The tracking technique is used to study the variations of vessel diameter in the direction of the vessel longitude axis. In addition, Tison et al. [16] have introduced a method to estimate the blood vessel diameter using active contours. The system proposed consists of a segmentation procedure which uses two active contours to detect the blood vessel outline and an approach to measure blood vessel diameters as the distance between two points of the edge. Lowell et al. [17] have proposed an algorithm to measure the vessel diameter to subpixel accuracy. This method is based on a two-dimensional difference of Gaussian model, where the model is optimized to fit a two-dimensional intensity vessel segment. Our proposed algorithm improves the performance of the measurement results compared with the works described above.

This paper is organized as follows: Section 2 illustrates the methodology of the proposed problem that has driven our research. Next, we briefly describe the datasets and the principal characteristics of the retinal image. Thereafter, we describe the method to convert the image from the space gray to binary image in order to introduce a thinning algorithm to get a 1-pixel-wide skeleton representing the center line of the vessel tree. The next step consists of a pixel classification algorithm which is dedicated to extracting the pixel information. We also try to explore the essentials of the Douglas-Peucker method used to determine the characteristic points. Afterward, we describe the detection of the contour using the snake method and a brief survey of his existing equations. Then, we illustrate the implementation of the proposed algorithm. The experiments applied on retinal images are explained step by step and the results are presented in Section 3. In Section 4, we discuss the properties of the obtained results. Finally, Section 5 concludes the paper by summarizing the proposed method.

## 2. Material and Methods

### 2.1. Proposed Method

The most common diseases of the eye and its related structures are diabetic retinopathy, hypertensive retinopathy, and macular degeneration. Diabetic retinopathy is a disease that happens due to damage in the retina blood vessels for people who have type 1 or 2 diabetes [1,2,3,4,5,6,7]. In addition, high blood pressure can induce harm to retina blood vessels and they can become narrow, limiting the function of the retina, and putting pressure on the optic nerve, causing vision problems. Macular degeneration occurs when abnormal blood vessels develop under the retina and macula; the condition can make blood vessels bleed or leak fluid. We can notice that these diseases have a direct consequence on the regional anatomy of the human retinal blood vessel. In this research study, we are interested in developing an automatic diagnosis system of the changes in levels of the human retina topology that can help the ophthalmologist to diagnose the patient [11,12,13,14,15,16,17,18,19,20,21,22].

**Figure 1:**
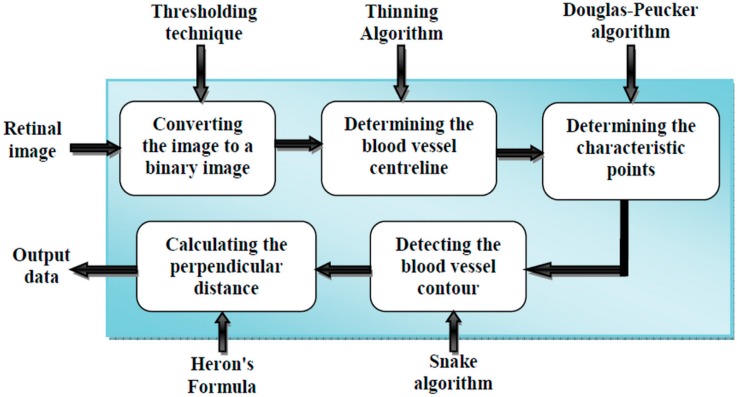
Block diagram of the proposed method.

The aim of this paper is to measure the diameter of blood vessels in retinal images. Figure 1 illustrates the different steps of our approach.

(i) First, we will give the image source and introduce its features.
(ii) Then, we will present pre-processing of the 2D image.
(iii) Subsequently, the determination of the control point using the Douglas-Peucker algorithm will be explained.
(iv) Then, we will detect the blood vessel contour using the active contour technique.
(v) Finally, the Heron’s formula will be presented to determine the blood vessels diameter.

### 2.2. Retinal Image Segmentation

#### 2.2.1. Image Source

There are several retinal images databases available. For example, one of the most used databases for vessel segmentation is the STARE (STructured Analysis of the Retina) database [23], which contains low-resolution images. On the other hand, there are other databases with higher resolution such as High-Resolution Fundus (HRF) Image Database [24].

##### STARE Database

**Figure 2:**
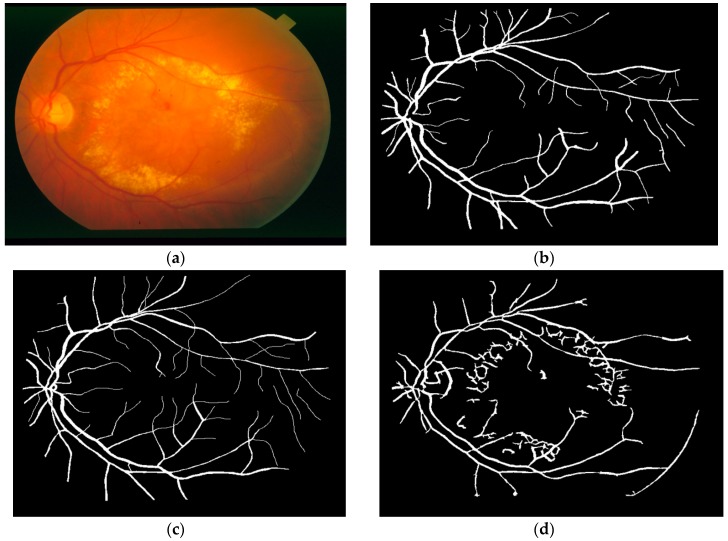
Example of a fundus image used: (**a**) Example of a raw image im0002.jpg; (**b**) Vessel networks marked by hand provided by Adam Hoover; (**c**) Vessel networks marked by hand provided by Valentina Kuznetsova; (**d**) Vessel networks produced by matched spatial filter probing algorithm.

In 1975, Michael Goldbaum, M.D. created and initialized the STARE Project, at the University of California, San Diego [23]. This project provides a full set of 400 retinal raw clinical images. Four sets of images are made usable, one set of 20 raw images was captured by a Topcon TRV-50 fundus camera with a 35 degree field of view; the slides that were produced were digitized in order to produce images with 700 × 605 pixel resolution with 24-bits per pixel (Figure 2a). The two other sets of 20 images (set 2 and 3) come with an additional vessel segmentation that was hand labeled—the first set was provided by Adam Hoover (Figure 2b) and the second set of images was provided by Valentina Kouznetsova (Figure 2c). The fourth set provides sample results as produced by the matched spatial filter probing algorithm (Figure 2d) [25].

##### High-Resolution Fundus (HRF) Image Database

**Figure 3:**
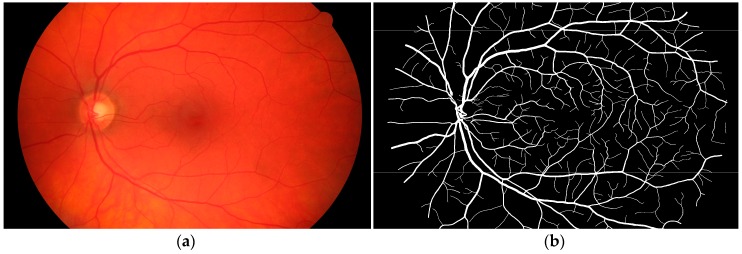
Example of a fundus image from the proposed database: (**a**) Example of a raw image im0002.jpg; (**b**) The manual segmentation of the vessels.

This database contains 45 fundus images. These images are divided into three sets: 15 images of healthy patients, 15 images of patients with diabetic retinopathy, and 15 images of glaucomatous patients [24]. All of these images were taken using a CANON CF-60UVi camera, the image's dimensions for the JPEG still images shooting are 3504 × 2336 pixels. Also, manual labeling of the vessels was done by experts in vessel segmentation and binary vessel segmentation images are available for each image. Figure 3a shows an example of a fundus image from the High-Resolution Fundus (HRF) Image Database with the corresponding manual segmentation (Figure 3b).

#### 2.2.2. Binary and Skeletonization Image

The next step of our work is to convert pictures into a binary image [26,27,28,29]. The binarization process converts a pixel code to multiple bits of 4, 8, or more on a single bit. The most used method is the thresholding technique [30,31]:

(1) $$I_{B}\left(i,j\right)=\{\begin{array}{c} 0\;if\;S>I_{E}\left(i,j\right)\\ 1\;if\;S<I_{E}\left(i,j\right) \end{array}$$

where *I*_E_ is the input image and *I_B_* is the binary image.

The blood vessel skeleton allows the complexity of the form to be reduced by finding the optimal curve. Thinning algorithms are typically used due to their effectiveness and reliability. The thinning algorithm is applied by removing all the points of the image contour except points that belong to the skeleton, which are left [32,33,34]. We can classify this algorithm as an iterative algorithm which erodes the outer pixels layers until there are no more layers that may be removed [35,36].

This technique does not take into consideration the blood vessel width, but could measure other qualities such as orientation, vessel segments length, and the presence of shortcomings, in addition to endpoint and bifurcation point detections.

#### 2.2.3. Point Detection

**Figure 4:**
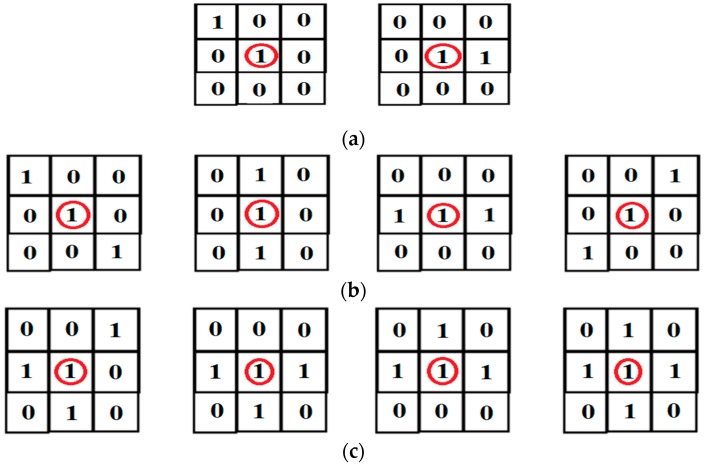
Pixel classification (the red circle represents the active pixels): (**a**) Endpoint; (**b**) Inner point; (**c**) Bifurcations point.

In our strategy, we apply a 3 × 3 sliding mask for determination of the endpoint and bifurcations points and we compute the number of the active neighboring pixels. If the center of the mask is an active pixel and the number of active neighboring pixels is 1, this point is the endpoint (Figure 4a). If there are two active neighboring pixels, this indicates an interior point (Figure 4b). Otherwise, if the number of active neighboring pixels is greater than 2, this indicates that the point is the bifurcations point (Figure 4c) [37,38].

#### 2.2.4. Determination of the Characteristic Points from the Blood Vessel Curve

The objective of the simplified polynomials algorithms is to simplify the lines by removing the extraneous bends while maintaining their overall shape and retaining the essential points of their shape, which are called characteristic points. There are several algorithms dedicated to accomplishing this goal, such as nth point elimination, a normal routine, and the Douglas-Peucker algorithm [39,40,41,42].

White [39] has conducted a study on these three simplified algorithms based on Marin’s work [40]. He showed that the results produced by the Douglas-Peucker algorithm are the best examples of the original lines representations in 86% of all tests. Thus, this algorithm was adopted in our work.

##### The Douglas-Peucker algorithm

**Figure 5:**
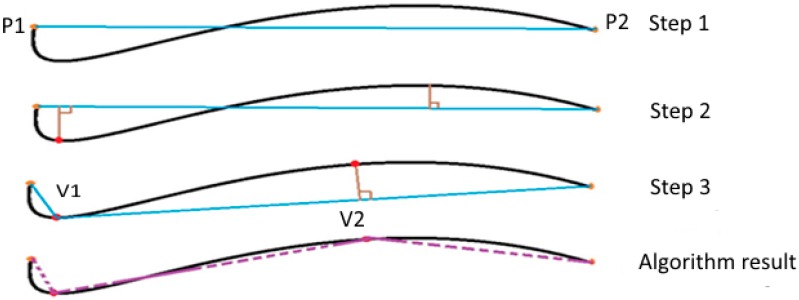
The different stages of the Douglas-Peucker algorithm.

The first step of the Douglas-Peucker algorithm is to connect the end nodes of a curve with a segment P1P2 (step 1 in Figure 5). The perpendicular distance between each curve point and the segment P1P2 is measured (step 2 in Figure 5):

If the points have a lower orthogonal distance than a defined tolerance value, then these points will be eliminated.

If not, the curve is divided by the most distant point from the P1P2 segment. This has the effect of creating two new curves V1 and V2 (step 3 in Figure 5). This process is repeated until all the distances become smaller than a tolerance value [41,42].

### 2.3. Edge Detection with Snake Algorithm

The snake algorithm was proposed by Kass et al. in 1988 [43]. The idea is to move the points to bring them closer to high gradient areas while maintaining the characteristics such as the contour curvature of the point distributions or other restrictions on the order of the points [44,45,46]. This algorithm begins by arranging the contour evenly around the object. Subsequently, the contour has two paths: either shrink or develop (it is located inside an object at the beginning of the algorithm) and try to scan the entire object shape to have the best forms. The algorithm tries to find a better contour position in order to minimize deviations from the constraints used [43]. The algorithm stops when it is not possible to improve or simplify the implementation, when the maximum iterations number is achieved.

#### 2.3.1. The Snake Algorithm

The snake model is described as a model of controlled continuity under the influence of the image forces [42]. The internal forces control the bending line characteristics, whilst the image forces serve to push the snake to detect the image characteristics. The snake position can be represented by the function *v*(*s*) = (*x* (*s*), *y* (*s*)) and can have its energy function written as follows:

(2) $$E_{\mathrm{Snake}}^{*}=\int_{0}^{1}E_{\mathrm{Snake}}\;v\left(s\right)\mathrm{dt}=\int_{0}^{1}E_{\mathrm{int}}\left(v\left(s\right)\right)+E_{\mathrm{ext}}\left(v\left(s\right)\right)$$

where *E*_int_ is internal energy given in equation (3), and *E*_ext_ is the external energy given in Equation (4).

#### 2.3.2. The Internal Energy

The internal energy is given in the following equation [42]:

(3) $$E_{\mathrm{int}}=E_{\mathrm{cont}}+E_{\mathrm{curv}}=\alpha \left(s\right){\left|V_{s}\left(s\right)\right|}^{2}+\beta \left(s\right){\left|V_{ss}\left(s\right)\right|}^{2}$$

where:

*E*_cont_: is the continuity energy, it ensures that the parameterization of the points remains equidistant from each other.
*E*_curv_: is the curvature energy, it maintains the rigidity of the snake.
v_s_(s): is the first derivative of screws with respect to s.
v_ss_(s): is the second derivative of v(s) with respect to s.
α(s): is the contour elasticity.
β(s): is the contour rigidity.

#### 2.3.3. The External Energy

The external energy is taken to be the gradient magnitude of the image and it is given in the following equation:

(4) $$E_{ext}=-\gamma {\left|\nabla G_{\sigma }\left(x,y\right)\times I\left(x,y\right)\right|}^{2}$$

where:

γ: is a constant used to control the importance of the *E*_ext_.
G_σ_(x,y): is a Gaussian kernel with a scale σ.

### 2.4. Methods

#### 2.4.1. Initialization.

The developed algorithm aims to support this method in three steps: The first step carried out the characteristic point detection using the Douglas-Peucker algorithm [41]. The process of this algorithm begins by plotting a line which is the linear regression for all points. This was followed by calculating the farthest point of the straight line of regression in order to calculate the new different regressions from left to right. This process will be repeated until getting an error smaller than a fixed limit. The next step will be devoted to the detection of blood vessel contour using the snake method, which is used to place an initial contour line around the shape to be detected [43]. This line will gradually deform according to the action of several forces that will pull or push it towards the shape. This dynamic is based on the notion of internal and external energy, the aim therefore being to minimize the total energy present along the curve. Finally, Heron’s formula should be applied in order to detect the blood vessel diameter. This step is detailed in the next section.

#### 2.4.2. Determination of Vessel Diameters

**Figure 6:**
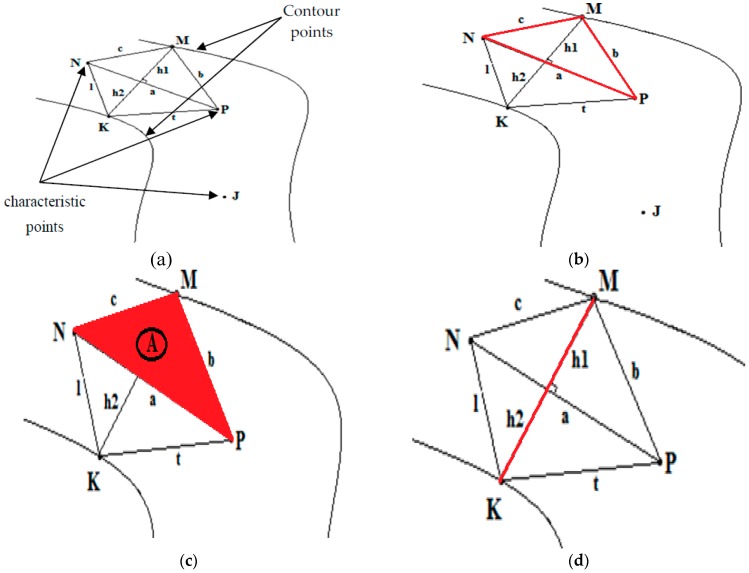
Technique used for the determination of the blood vessel diameters: (**a**) Presentation of the basic elements used (the two black arrows represents the Contour points and the three other black arrows represents the characteristic points); (**b**) Find the perimeter of a triangle; (**c**) Calculate the area of a triangle; (**d**) Calculate the heights of the triangles and the blood vessel diameters.

The Douglas-Peucker algorithm aims to determine the characteristic points, we then use the snake method to detect the blood vessels contour. These characteristic and contour points are used to calculate the blood vessel diameters. The computation process of the proposed algorithm is shown in Figure 6.

Step 1: Connect the first characteristic point (*N*) and the second characteristic point (*P*) in the same blood vessel branch to a straight line $\bar{\mathrm{NP}}$. Then, we connect these two characteristic points at any chosen contour point that belongs to the same blood vessel branch ($\bar{\mathrm{NM}}$ and $\bar{\mathrm{MP}}$) (see Figure 6a).

Step 2: We calculate the Euclidian distance of the three lines with the following equations:

$$a=\sqrt{{\left(x_{N}-x_{P}\right)}^{2}+{\left(y_{N}-y_{P}\right)}^{2}}$$

$$b=\sqrt{{\left(x_{M}-x_{P}\right)}^{2}+{\left(y_{M}-y_{P}\right)}^{2}}$$

$$c=\sqrt{{\left(x_{N}-x_{M}\right)}^{2}+{\left(y_{N}-y_{M}\right)}^{2}}$$

Step 3: We calculate the heights of the different triangles *MNP* (*h*1) and KPP (*h*2), and the sum of these two heights in order to get the diameter of the blood vessel (see Figure 6d):

We can use Heron’s formula to find the area of a triangle using the three side lengths:

*M*, *N*, and *P*, the three vertices of the triangle, and *a*, *b*, and *c* the three sides respectively opposite to the three vertices. Then, we set $S=\frac{a+b+c}{2}$ (half perimeter) (see Figure 6b).

There is a formula (Equation (5)) to calculate the area of triangle (called A) (see Figure 6c):

(5) $$A=\sqrt{S\left(S-a\right)\left(S-b\right)\left(S-c\right)}$$

Also, we can note that the area is given by the following formula (Equation (6)):

(6) $$A=\frac{h\times a}{2}$$

Then, we can deduce:

$$\sqrt{S\left(S-a\right)\left(S-b\right)\left(S-c\right)}=\frac{h_{i}\times a}{2}\;\mathrm{With}\;i\;=1,\;2.$$

From where:

$$h_{i}=\frac{2\times A}{a}$$

## 3. Results

The system was implemented on a personal computer (PC) with an Intel Pentium B960 CPU @ 2.20 GHz and 4 GB of RAM. The program was implemented in MATLAB^®^ 2014b and the results were also graphed with MATLAB^®^.

### 3.1. Segmentation of the Human Retina Image

**Figure 7:**
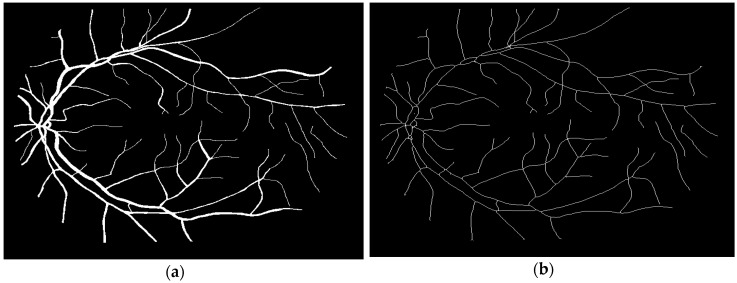
Binarization and skeletonization image: (**a**) Binary image; (**b**) Skeletonization image.

The determination of the blood vessel diameter using image analysis necessitates the initial segmentation of the retinal image so as to produce binary images. The typical path of making a binary image (Figure 7a) from a greyscale image (Figure 2b) is made by the thresholding method. Subsequently, the skeleton of the blood vessels is produced from the input binary image by the thinning process. This is shown in Figure 7b.

### 3.2. Douglas-Peucker Algorithm

**Figure 8:**
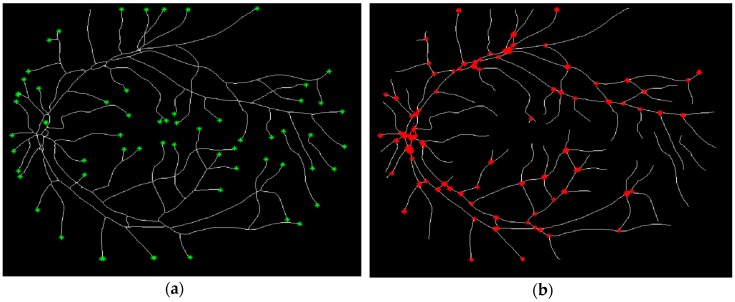
Detection of the endpoints and bifurcation points: (**a**) Endpoints; (**b**) Bifurcation points.

To run the Douglas-Peucker algorithm, we began by determining the trajectory curve of each vessel. To accomplish this goal, we tried to detect the endpoints (Figure 8a), the bifurcation points (Figure 8b), and the internal points in the retinal image.

**Figure 9:**
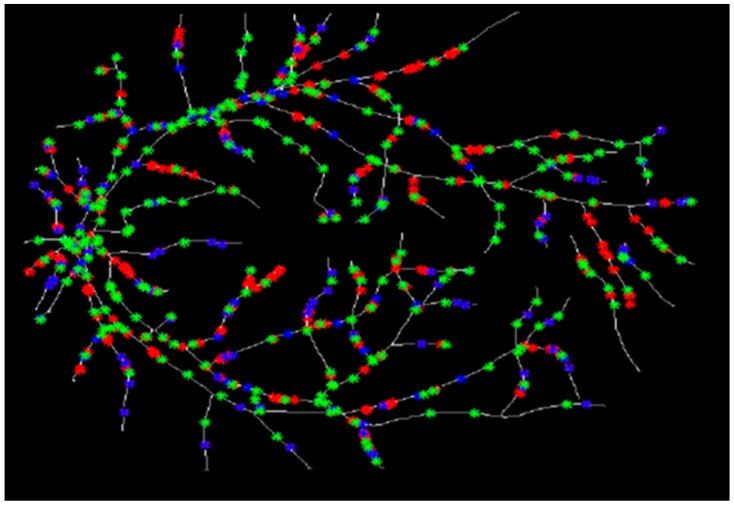
Determination of the characteristic points for three various values of ε. The red points show the characteristic points for *ε* = 0.5, the blue points depict the characteristic points for *ε* = 1, and the green points present the characteristic points for *ε* = 1.

The Douglas-Peucker algorithm reduces the data storage amount and the number of points from a given line, while maintaining its principal geometrical and topological properties. The data compression ratio (DCR) (Figure 9) is defined as follows:

DCR=(N−n)n v

**Figure 10:**
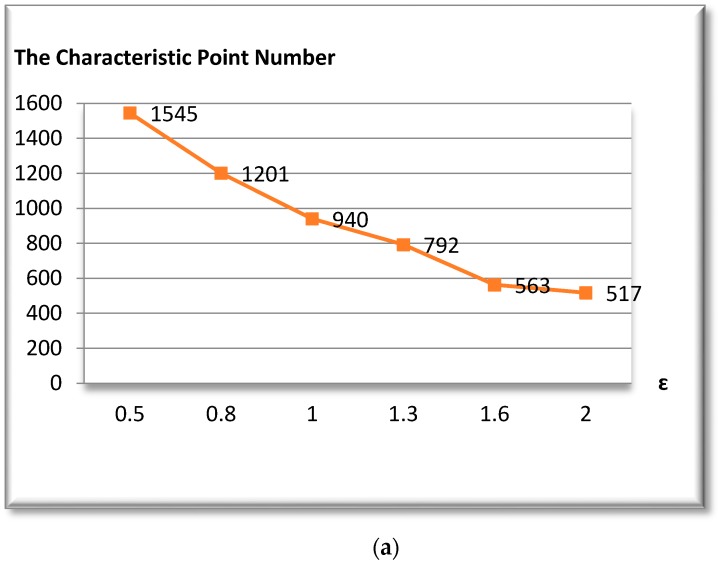

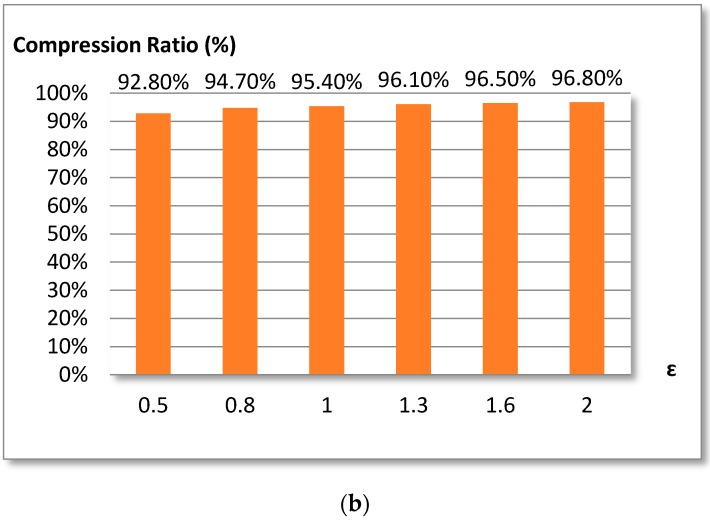
(**a**) The characteristic point number; (**b**) The compression ratio of the vascular tree.

Figure 10a shows the number of characteristic points obtained for different tolerances values *ε*. We notice from this figure that the characteristic point number is between 1545 for *ε* = 2 and 517 for *ε* = 0.5, and Figure 10b shows that the compression ratio is around 92% for *ε* = 0.5, and it can be improved to 96.6% for *ε* = 2. It should be observed that the compression ratio and the characteristic point number vary according to the value of *ε*. If *ε* is high, the number points detected is low, and vice versa.

### 3.3. Diameter Measurement

**Figure 11:**
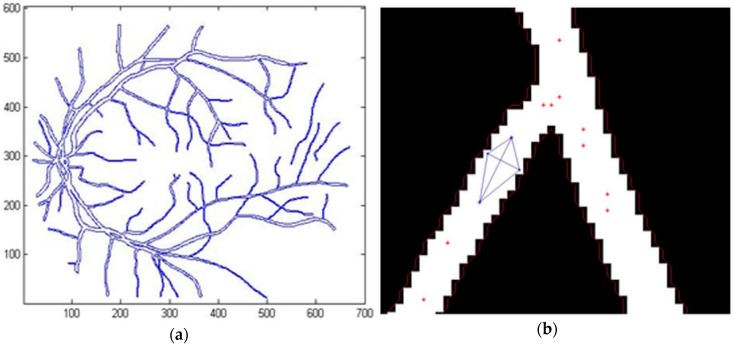
Calculation of the blood vessel diameter: (**a**) Contour detection; (**b**) Image depicting the calculation of the blood vessel diameter (the blue lines represents the triangles detection and the red dots represents the characteristic points).

Figure 11a shows an example of contour detection by the active contours method. Figure 11b shows the characteristic point determination and represents the method used to calculate the blood vessel diameter.

To accomplish the goal of this research project, two databases were chosen in order to ensure the performance of the proposed algorithm. Three images were used for each database, the STARE database which is illustrated in Table 1 and the HRF database, which is shown in Table 2.

The appropriate method to test and compare our measurement technique is to compare the same blood vessel measurements. To this end, we tested the accuracy of the algorithm presented using 30 widths obtained from six fundus images. Thus, in order to evaluate the effectiveness of our proposed program and also to obtain a more reliable analysis of the experimental data, we calculated the mean and standard deviation (SD) [47,48] difference between the manual measurement and the measurement using the proposed method and noted here *E*_avg_ and SD respectively, as shown in the two tables below.

The standard deviation (SD) estimate is defined by the following formula [47,48]:

$$SD=\sqrt{\frac{\sum {\left|x-\bar{x}\right|}^{2}}{n}}$$

The mean value (*E*_avg_) is given by the following equation [47]:

$$\mathrm{Eavg}=\frac{\sum \left|x-\bar{x}\right|}{n}$$

where:

- *x* is the manual measurement value.
- $\bar{x}$ is the measured value obtained using the proposed method.
- *n* is the number of test measurements.

Table 1 and Table 2 provide a comparison of the accuracy between the manual measurements and the proposed method. This comparison is made respectively to the STARE Database [23] and High-Resolution Fundus (HRF) Image Database [24].

Column 5 in Table 1 and Table 2 shows the difference between the manual measurements and the diameter measurements obtained by the proposed method. Columns 6 and 7 are the standard deviation (SD) and mean (*E*_avg_) of the difference between the types of measurements, respectively. According to both Table 1 and Table 2, among the two datasets, the proposed method obtained the highest performance on the images from the High-Resolution Fundus (HRF) Image Database with the mean difference between 0.009 and 0.03 and standard deviation between 0.0063 and 0.0234. The test using images from the STARE Database resulted in mean and standard deviation differences that were between 0.0211 and 0.2752 and 0.0162 and 0.1401, respectively—these results are slightly higher than the results mentioned above.

## 4. Discussion

The numerical results obtained by this study are compared with the values resulting from the formulations available in the literature.

Kumar et al. [11] tested their method on four sets of data, and their proposed method had the following performance: the Kick-Point Image Set (KPIS)database resulted in mean and SD difference values of 0.50 and 0.60, respectively; the central light reflex validation test using the High Resolution Image Set (HRIS) database resulted in mean and SD difference values of −0.55 and 1.79, respectively; the test using the Central Light Reflex Image Set (CLRIS) database result in mean and SD difference values of 0.21 and 0.79, respectively; and tests using noisy pathological images (Vascular Disease Image Set (VDIS)dataset) resulted in mean and SD difference values of −0.64 and 1.18, respectively. In Lowell et al. [17], the standard deviation difference generated by his method ranged between 0.124 and 0.464 pixels. On the other hand, Zhou et al. [19] reported that their approach based on the Gaussian 1-D model, had a standard deviation (SD) value of the order of 0.58. Add to that, the method of Chapman et al. [18] resulted in a mean difference between manually measured diameters and their test method in red-free images of about 3 pixels, and a standard deviation of 1.40 pixels. Gao et al. [15] provided blood vessel diameter measurements from a paired fluoresce in a 259 red-free image. The error generated by this technique was −0.9 for the mean difference value and 1.6 for the SD difference value, the error in this case is even larger. However, our results show that the maximum obtained difference does not exceed 0.3085 in absolute value. Moreover, the results we obtained are very consistent, with a small difference between the proposed method and manual method. Our technique has a standard deviation (SD) difference ranging between 0.0092 and 0.1401 pixels and produced a smaller mean difference value between 0.0090 and 0.2752 pixels.

We can notice that the presented algorithm is five times more accurate than the techniques proposed by Kumar et al. [11], and three times more precise than the method of Lowell et al. [17] and Zhou et al. [19]. We can also add, the obtained results from 30 measurement samples show that the proposed algorithm is over 70% more accurate than the other techniques.

Note that our proposed method provides information on the blood vessels in the most reliable and reproducible manner, and that it can be used to automatically diagnose the retinal blood vessels.

Another advantage is that the developed system requires less time because it has an execution time that is less than 2 s. In addition, the results also show that this method appears to demonstrate good accuracy for all the databases.

The importance of this work lies in the fact that it can determine the morphological changes that target the human retinal blood vessels, and subsequently, it helps to facilitate several tasks such as diagnosing diseases during a clinical examination of the human retinal image and monitoring progress in vessels.

## 5. Conclusions

This objective of this study was mainly centered upon creating an automated computer system for measuring the blood vessel diameters of the human retina from an ocular fundus image. Several algorithms were used, including those for converting the image to a binary image to facilitate the determination of the bifurcations location, the endpoints, and the inner point to detect the vessel blood curves. Then, we determined the blood vessel contour using the active contour method. The next step which is described in this work was the determination of characteristic points by the Douglas-Peucker algorithm.

The final algorithm was used to calculate the diameter of the vessel. We presented a set of comparisons related to the vessel width between a manual measurement and the proposed computerized system. The obtained results are three times more precise than the other methods and over 70% more accurate than the other techniques. It can be noted that our developed system requires less time and provides more reliable and reproducible information regarding the morphology of blood vessels.

## Figures and Tables

**Table 1:** Comparison of the diameter measurements (in pixels) between the proposed method and manual method for the STARE Database.

Image	Vessel No	Diameter (Manual)	Diameter (Proposed Method)	Difference	Standard Deviation (SD)	Average (*E*_avg_)
im0002.ah.jpg	1	6.6212	6.6436	−0.0224	0.0162	0.0211
	2	5.3101	5.3099	0.0002		
	3	4.4721	4.4721	0		
	4	9.2540	9.2487	0.0053		
	5	13.6255	13.7002	−0.0777		
im0139.ah.jpg	1	4.0000	3.752	0.2480	0.1094	0.2390
	2	4.1231	4.3872	−0.2641		
	3	5.8310	5.642	0.189		
	4	8.0000	8.176	−0.176		
	5	3.0000	2.682	0.318		
im0077.ah.jpg	1	7.8102	7.9394	−0.1292	0.1401	0.2752
	2	4.2426	4.4792	−0.2366		
	3	2.8284	2.674	0.1544		
	4	3.1623	2.6152	0.5471		
	5	8.9443	9.2528	−0.3085		

**Table 2:** Comparison of the diameter measurements (in pixels) between the proposed method and manual method for the High-Resolution Fundus (HRF) Image Database.

Image	Vessel No	Diameter (Manual)	Diameter (Proposed Method)	Difference	Standard Deviation (SD)	Average (*E*_avg_)
10_h.tif	1	18.3848	18.3564	0.0284	0.0234	0.0307
	2	5.000	4.887	0.1130		
	3	10.0499	10.0535	−0.0036		
	4	2.8284	2.8301	−0.0017		
	5	2.5381	2.5312	0.0069		
02_h.tif	1	3.0000	2.9654	0.0346	0.0092	0.0161
	2	15.0000	15.028	−0.0280		
	3	28.4253	28.4198	0.0055		
	4	20.0000	20.0035	−0.0035		
	5	9.2195	9.2105	0.0090		
15_h.tif	1	22.6274	22.6248	0.0026	0.0063	0.0090
	2	12.0000	12.0056	−0.0056		
	3	21.0950	21.1256	−0.0306		
	4	5.8310	5.8296	0.0014		
	5	7.6158	7.6205	−0.0047		
